# PLK-1 Targeted Inhibitors and Their Potential against Tumorigenesis

**DOI:** 10.1155/2015/705745

**Published:** 2015-10-18

**Authors:** Shiv Kumar, Jaebong Kim

**Affiliations:** Department of Biochemistry, Institute of Cell Differentiation and Aging, College of Medicine, Hallym University, Chuncheon, Gangwon-do 200-702, Republic of Korea

## Abstract

Mitotic kinases are the key components of the cell cycle machinery and play vital roles in cell cycle progression. PLK-1 (Polo-like kinase-1) is a crucial mitotic protein kinase that plays an essential role in both the onset of G2/M transition and cytokinesis. The overexpression of PLK-1 is strongly correlated with a wide spectrum of human cancers and poor prognosis. The (si)RNA-mediated depletion of PLK-1 arrests tumor growth and triggers apoptosis in cancer cells without affecting normal cells. Therefore, PLK-1 has been selected as an attractive anticancer therapeutic drug target. Some small molecules have been discovered to target the catalytic and noncatalytic domains of PLK-1. These domains regulate the catalytic activation and subcellular localization of PLK-1. However, while PLK-1 inhibitors block tumor growth, they have been shown to cause severe adverse complications, such as toxicity, neutropenia, and bone marrow suppression during clinical trials, due to a lack of selectivity and specificity within the human kinome. To minimize these toxicities, inhibitors should be tested against all protein kinases *in vivo* and *in vitro* to enhance selectivity and specificity against targets. Here, we discuss the potency and selectivity of PLK-1-targeted inhibitors and their molecular interactions with PLK-1 domains.

## 1. Introduction

**Figure 1:**
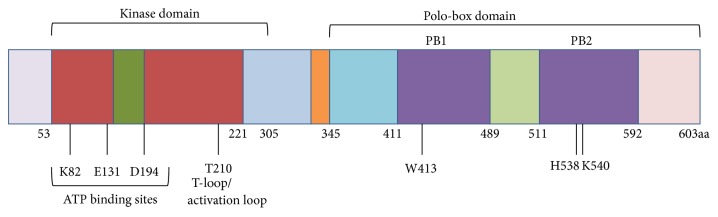
PLK-1 polypeptide sequence of human. PLK-1 gene encodes a polypeptide sequences with 603 amino acids. PLK-1 consists of two types of domains (1) the conserved ser/thr N-terminal kinase domain (53-305aa). There are three ATP-binding cassettes in kinase domain: Lys82, Glu131, and Asp194, responsible for ATP-binding and T-loop (Thr210). (2) Two C-terminal polo-box domain (411-592aa), three key residues at PBD: Trp414, His538, and Lys540 are responsible for phosphopeptide binding.

Polo-like kinase-1 (PLK-1), a serine/threonine (Ser/Thr) protein kinase, is highly conserved from yeast to humans and is reported to play a role in the mitotic events of the fruit fly. Five PLK family members are known in humans: PLK-1, -2, -3, -4, and -5 [1, 2]. All members contain an N-terminal ATP-binding catalytic domain known as the kinase domain, and two C-terminal noncatalytic domains known as the Polo-box domains (PBDs) as shown in Figure 1 [3]. In contrast, PLK-4 contains only one C-terminal PBD domain, whereas PLK-5 has no N-terminal catalytic domain. In addition, PLK-5 is a distantly related member of the PLK family and exhibits different functions as well as a different tissue distribution. PLK-5 plays a role in neurobiology and DNA damage response [4, 5]. In mitotic phase, Aurora-A-Borealis phosphorylates the T210-loop to activate PLK-1. Thus, PLK-1 interacts with PBD-bound substrate and progresses the cell cycle. PBD also plays a pivotal role in the subcellular localization and substrate interaction of PLK-1. PLK-1 is the most characterized member of PLK family because of its strong association with many regulatory events progressing during mitosis, such as G2/M transition, spindle assembly maturation, chromosome segregation, and mitotic exit [6, 7]. Therefore, PLK-1 is one of the key players in mitosis, ensuring the proper regulation of G2/M onset; nevertheless, the deregulation of PLK-1 leads to multiple defects in metaphase, termed mitotic defects, and favors the promotion of aberrant cell survival. These defects lead to aneuploidy and genomic instability within the cells and cause tumorigenesis (aneuploidy, a hallmark of cancer) [8]. Furthermore, the overexpression of PLK-1 is strongly associated with many types of human cancers because upregulated PLK-1 causes the inactivation and/or degradation of tumor suppressor gene p53 in a G2-and S-phase-expressed1 (GTSE1) and Topo-1 binding protein- (TOPORS-) dependent manner, respectively [9, 10]. Moreover, in the absence of p53, the ATR-ATM checkpoint machinery fails to recognize DNA damage, causing cells to enter mitotic phase with a high load of genomic defects. In addition, the overexpression of PLK-1 inactivates CDK-1 in a CDC25C-dependent manner and triggers tumorigenesis [11]. Many studies have documented that PLK-1 is considered a mitotic proto-oncogene [12]. A wide range of human cancers have been screened to validate PLK-1 inhibition during tumor growth. Thus, the inhibition of PLK-1, negatively affects cancer cell proliferation and reduces tumor growth [11, 13–17]. Furthermore, many studies have proven that overexpression of PLK-1 is not only related to tumorigenesis but also highlighted in the poor prognosis of cancer [18, 19]. Additionally, numerous studies have been published examining the inhibitory potential of PLK-1 as an antitumor drug target by using different approaches, including antisense oligonucleotides, small interfering (si) RNA and small molecules targeting the catalytic and/or PB domains* in-vivo* and* in-vitro* [20–22]. These studies suggested that PLK-1 is a promising, validated, attractive therapeutic drug target. These insights have initiated the design of various types of small molecules to downregulate and/or inhibit the overexpression of PLK-1 and regress tumor growth (Figure 4). PLK-1-targeted inhibitors are categorized into various classes on the basis of many properties such as the source of origin, biochemical properties, targeted domains, and interaction properties [23–29]. These inhibitors target PLK-1 as the Achilles heel of tumors [30, 31]. Although, most of the inhibitors show potent therapeutic capability in treating cancer, they also have a high risk of toxicity, owing to weak or no target selectivity against targeted domains because of the high similarity in the ATP-binding pocket and conformation. Therefore, PLK-1-targeted inhibitors are a point of emphasis for understanding the mechanism of action/s and interaction specificity with targeted residue/s. This knowledge will help reduce the toxicity level and increase the selectivity and specificity of the inhibitors to develop a safer, higher potency, and more selective drug-like molecules. Bioinformatics approaches have become an essential part of drug discovery to validate the efficacy and binding specificity of small molecules and to understand the mechanism of action against targets. These* in silico* tools help to improve the therapeutic value of the inhibitors and reduce their toxicity level to provide better therapeutic agents. In this study, we will discuss the potential, selectivity, and specificity profiling of PLK-1 targeted inhibitors against binding sites in an attempt to provide more selective and potent antitumor therapeutic agents.

*Classification*

*(a) Depend upon the Source*

- Natural source: Aristolactam AIIIa, Scytonemin, Wortmannin,
- Synthetic source: ON01910.Na, BI 2536, BI 6727, GSK461364A, HMN-176, SBE13, ZK-thiazolidinone (TAL), Compound 36, Compound 15, Compound 38, NMS-P937, LFM- A13, RO3280, TAK-960,
- Peptides: MQSpTPL or MAGPMQSpTPLNGAKK, LLCSpTPNG, PLHSpT,
- RNAi: TKM-080301.

*(b) Binding Site Based Classification*

1. Inhibitor Interaction with kinase domain:
  - Non ATP-competitive type: ON01910.Na, Cyclapolin 1,
  - ATP-competitive type: BI 2536, BI 6727, GSK-461364A, HMN-176, SBE13, ZK-thiazolidinone (TAL), Scytoemin, Wortmannin, Compound 36, Compound 15, Compound 38, NMS-P937, LFM-A13, RO3280, TAK-960,
  - RNAi: TKM-080301,
  - Others: DAP81, PHA680626.
2. Inhibitor Interaction with PBD domain:
  - Natural or semisynthetic: Poloxin, Poloxipan, Purpurogallin, Aristolochia AIIIa,
  - Peptide: MQSpTPL or MAGPMQSpTPLNGAKK, LLCSpTPNG, PLHSpT.

## 2. Targeting against the PLK-1 Kinase Domain

**Figure 2:**
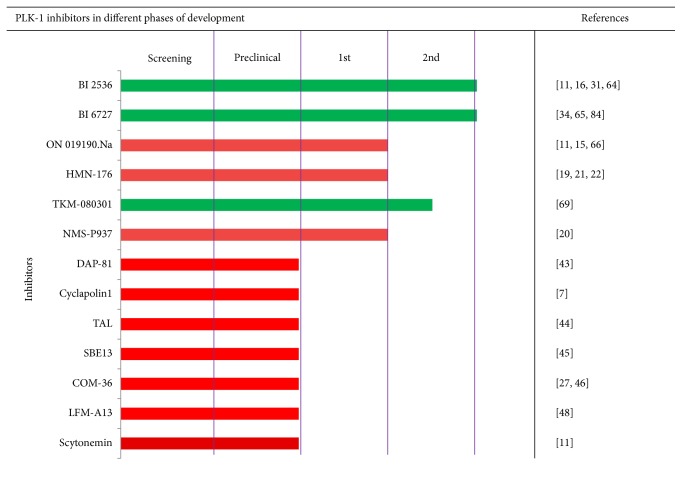
PLK-1 inhibitors are ongoing in different phase of clinical trials.

**Figure 4:**
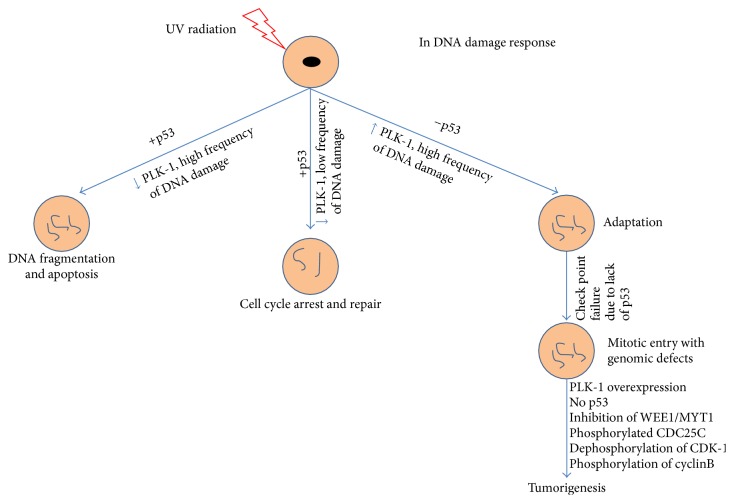
Role of PLK-1 in DNA damage based induction of tumorigenesis. In DNA damage response, overexpression of PLK1 degrades and/or inactivates the p53 in TOPORS and GTSE1 dependent manner (Figure 3). Consequently, cell enters in mitotic phase with high load of genomic defects. G2/M transition, PLK-1 dephosphorylates CDK-1 by activated CDC25C and also inhibits the CDK-1 activator WEE1/MYT1 to onset the mitotic entry with genomic defects and cause the tumorigenesis.

The protein kinases catalyze the transfer of the *γ*-phosphate group of ATP to substrates containing Ser/Thr/Tyr amino acid residues. Many studies have documented that the kinase domain is one of the most promising drug targets [27]. During the last decade, PLK-1 catalytic domain targeted inhibitors have been identified from the screening of natural and synthetic compound libraries. These inhibitors interfere with the catalytic activity of PLK-1 and diminish its expression. Many of the inhibitors are ongoing in different phases of clinical trials, shown in Figure 2, and show promising therapeutic value, but also exhibit poor selectivity and specificity to PLK-1. The catalytic cleft for a large group of protein kinases is not only highly conserved among kinases but also similar in sequence and conformation. Subsequently, inhibitor development after lead optimization with better selectivity against the kinase domain remains a significant challenge [27].

Structure-guided drug design is an improved strategy to design more selective and potent drug like molecules. Various groups of researchers have determined the crystal structure of the kinase domain. The first kinase domain was crystallized by using ankyrin repeat proteins (DARPins) to address the active confirmation [32]. Meanwhile, another study addressed the binding mechanism of wortmannin by using the cocrystal structure of zebrafish PLK-1 [33]. Furthermore, the crystal structure of the T-loop mutant T210V interacted with PLK-1 inhibitors, termed nonhydrolyzable ATP analog adenylyl imidodiphosphate (AMPPNP) and PHA-680626, at 2.4 and 2.1 Å resolutions, respectively [34]. Later, a structure-activity relationship study of BI 2536 indicated that a methoxy group is the main entity determining the specificity and selectivity of PLK-1 and non-PLK-1 kinases through interaction with Leu 132, which is present in a small pocket of the hinge region of PLK-1 [35]. In addition, NMS-P937, a potent ATP-mimetic inhibitor, inhibits the methylated crystals of a PLK-1_aa36-345_ construct [34, 35]. The kinase domain targeted inhibitor is described in Table 1.

### 2.1. Scytonemin

Initially, scytonemin was identified as a PLK-1 inhibitor and was isolated from many strains of Cyanobacteria,* Calothrix* sp., and* Lyngbya aestuarii* [1, 2]. The structure of scytonemin was determined in 1993 [3]. PLK-1 activates the M-phase inducer phosphatase 3 (CDC25C) by phosphorylation and leads to the CDC25C-dependent inhibition of WEE/MYT1 to initiate mitosis during the G2/M transition [4–6]. Scytonemin inhibits PLK-1 with an IC_50_ of 2 ± 0.1 *μ*M in a concentration-dependent manner* in vitro* and disrupts the activation of CDC25C [7, 8]. Another study has reported that scytonemin failed to inhibit PLK-1 up to IC_50_ of 3-4 *μ*M [12]. It was later found to be a nonselective inhibitor of PLK-1 due to the inhibition of other mitotic factors, including myelin transcription factor (MYT1), CDK-1, checkpoint kinase-1 (Chk-1), and protein kinase C (PKC), with a similar half-inhibitory concentration [7]. Scytonemin is currently undergoing preclinical trials.

### 2.2. ON01910.Na

ON018910.Na is a hydrophilic benzyl styryl sulphone analog that was first reported as a PLK-1 inhibitor that functions in an ATP-independent manner. ON01910.Na treated cells exhibited inhibition of the PI3K/AKT pathways and downregulation of cyclin D1 with activation of the apoptosis-related genes NOXA and BIM. Furthermore, another study demonstrated that ON01910.Na treated cells showed inhibition of phosphorylation of CDC25C, a downstream protein of cyclin B, and activated the caspase pathway by the downregulation of Bid, Bcl-xl and Mcl-1 in B-cell lymphoma [36, 37]. A wide range of cancer cell lines have been screened to evaluate the anticancer potential and selectivity of ON01910.Na. ON01910.Na-treated cells displayed effects on microtubule dynamics and caused mitotic defects such as multipolar spindles and centrosome abnormalities. As a result, ON01910.Na treated cells exhibited the mitotic arrest and spindle abnormalities and caused the apoptosis [16]. Moreover, ON01910.Na also inhibited drug-resistant cell lines with an IC_50_ ranging from 50 to 250 nM* in-vivo* and* in-vitro *[14]. However, an additional study demonstrated that ON01910.Na did not inhibit PLK-1 enzymatic activity up to a concentration of 30 *μ*M in an* in vitro* kinase assay and was unable to show RNAi-induced cellular phenotypic resemblance [15]. Furthermore, the clinical development phase I studies of ON01910.Na have been successfully completed with some common adverse effects, including fatigue, pain, nausea, vomiting, and abdominal pain [14]. Moreover, an additional phase I study has been completed against advanced cancers or B-cell chronic lymphocytic leukemia with mild toxicities, including abdominal, skeletal, and tumor pain, nausea, and fatigue with mild hematological toxicities. Furthermore, the phase I study recommended a 3120 mg dose for the phase II studies, and the pharmacokinetic study showed that the half-life of distribution and elimination is 1 hour and 27 hrs, respectively [38]. ON01910.Na is now undergoing phase III clinical trials for myelodysplastic syndromes (MDS) correlated with misexpression of cyclin D1 [37].

### 2.3. Wortmannin

Wortmannin is a steroid metabolite of the fungi* Penicillium funiculosum* and* Penicillium wortmannin* and was initially characterized as a phosphatidylinositol-3-kinase (PI3K) inhibitor. However, a later study observed that wortmannin also inhibited PLK-1 with an IC_50_ of 24 nM [17]. According to the crystal structure analysis, wortmannin interacts with the Lys68 residue of the PLK-1 kinase domain [13]. Furthermore, PLK-1 is also inhibited by wortmannin in a time-dependent manner without inhibiting the remaining PLKs family members. However, wortmannin also interacts with PLK-1/2/3 with a similar binding affinity (1.5–3.0 *μ*M) [18].

### 2.4. HMN-176

HMN-176 is an active metabolite of the synthetic antitumor oral prodrug, HMN-214, and was developed by the Japan-based company Nippon Shinyaku Co. Ltd. HMN-176 did not show direct catalytic inhibition of PLK-1. A wide range of human cancers have been screened to validate the potential of HMN-176 as an anticancer chemotherapeutic agent with an IC_50_ value of 118 nM* in vitro* [19]. HMN-176 interferes with the subcellular localization of PLK-1 in centrosome and cytoskeleton structure. Furthermore, HMN-176 also downregulates (multidrug resistant)* MDR1* by inhibiting the transcription factor Nuclear Transcription Factor Y subunit alpha (NF-Y) and inducing mitotic cell cycle arrest [21, 22]. HMN-176 showed potent activity against various human tumor xenografts (Table 3). Furthermore, a phase I pharmacokinetic study of HMN-176 demonstrated that the maximum tolerated dose (MTD) of HMN-176 (8 mg/m2/day) is well tolerated, with some modest adverse effects, including myalgia/bone pain syndrome, hyperglycemia, neutropenic sepsis, and neuropathy. The adverse effects depend on the oral dose schedule and the degree of treatment [21].

### 2.5. NMS-P937 1H-Pyrazolo(4,3-H)quinazoline-3-carboxamide,4,5-dihydro-1-(2-hydroxyethyl)-8-((5-(4-methyl-1-piperazinyl)-2 (trifluoromethoxy)phenyl)amino)

NMS-P937 is a derivative of pyrazolo quinazoline that was developed by Nerviano Medical Science, Milano, Italy. NMS-P937 is an oral available, selective ATP-competitive inhibitor [20]. NMS-P937 showed high selectivity among a panel of more than 250 protein kinases and no cross-reactivity with other PLK-1 family members. More than 100 cell lines related to hematological and solid cancer have been treated to evaluate the potential of NMS-P937, which has an inhibitory concentration less than 0.02 *μ*M. Another study has documented that NMS-P937 is well-tolerated with good potency and efficacy in preclinical xenograft tumor models. Moreover, the 2′-trifluoro-methoxy moiety of NMS-P937 determined the molecular selectivity for PLK-1 and the rest of the kinases, and the 2′-trifluoro-methoxy moiety fits in the ATP-binding pocketsformed by Arg57 and the hinge residues Leu132-Cys133-Arg134 [23]. NMS-P937 has been validated as an anticancer agent against different preclinical rodent, and nonrodent models of acute myelogenous leukemia (AML), exhibiting inhibition of PLK-1-mediated phosphorylation of TCTP at Ser46 and triggering the apoptosis induction. NMS-P937 also showed good oral bio-availability upon the combination with the white blood cell cancer drug Cytarabine and prolonged survival [29]. Furthermore, a phase I dose-escalation against advanced or metastatic solid tumors study has been completed successfully (https://clinicaltrials.gov/ct2/show/record/NCT01014429).

### 2.6. GSK461364A

GSK461364A is a selective thiophene amide derivative PLK-1 inhibitor that was designed by Glaxo Smith Kline [26]. It is an ATP-competitive inhibitor. GSK461364A has been screened against more than 120 cancer cell lines to validate its chemotherapeutic potency and selectivity. It showed at least 400-fold greater inhibitory potential for PLK-1 over other family members, including PLK-2/3, with an IC_50_ value of 50 nM. Moreover, GSK461364A was also screened against a panel of 260 protein kinases and exhibited an IC_50_ value of <1 *μ*M against only 10 additional kinases [24, 25]. GSK461364A-treated cells predominantly showed metaphase arrest with polo spindle-like appearance [26]. Additionally, GSK461364A showed not only* in vitro* inhibition of PLK-1 but also dose-dependent tumor growth inhibition in various established tumor xenografts. Moreover, GSK461364A also prevented brain metastases from breast cancer. This study suggests that PLK-1 may be a therapeutic target for the inhibition of metastases [27]. GSK461364A is a well-tolerated inhibitor in phase I studies against advanced solid tumors on non-Hodgkin's lymphoma (excluding HIV-associated lymphoma) with minimal risk of toxicity, such as pulmonary embolism and neutropenia. It showed different dose-limiting toxicity profiles during phase I studies and neutropenia at a dose of 100 mg, whereas at a dose of 225 mg pulmonary embolism or sepsis was observed [28]. GSK461364A is no longer in clinical trials for development.

### 2.7. BI 2536

BI 2536 is a dihydropteridinone derivative that was developed by Boehringer Ingelheim. BI 2536 is the most potent ATP-competitive type inhibitor of PLK-1, arresting a large number of cell lines in the G2/M phase with mitotic abnormalities, such as the monopolar spindle and aberrant accumulation of cyclin B1 [16, 31]. BI 2536 inhibits PLK-1 with an IC_50_ of 1 nM* in vitro*. Nonetheless, it also showed activity against other members of the PLK family (except PLK-4 and 5) at different concentrations, PLK-2 and PLK-3 with IC_50_ values of 3.5–4.0 nM and 9 nM, respectively. Moreover, BI 2536 showed 1000-fold better selectivity against a panel of tyrosine and serine/threonine kinases [16]. BI 2536 is a well-tolerated inhibitor with potent efficacy* in vivo*, and it reduced tumor size in several well-established xenograft models [31]. In addition, BI 2536 is well tolerated at the MTD with 200 mg/week/cycle or when used twice a week at 100 mg/cycle, showing reversible dose-limiting toxicity, namely, neutropenia during phase I studies. Additionally, neutropenia is a reversible complication with the combination of nutlin (nutlin-3) and BI 2536. Nutlin is a class of MDM2 binding molecules that stabilizes p53 without affecting the efficacy of BI 2536 [30, 32]. In a phase II clinical trial, patients with specific cancers including non-small cell lung, pancreatic, or hormone-refractory prostate cancer were included for further study and showed grade 3-4 adverse effects, such as neutropenia, thrombocytopenia, febrile neutropenia, anemia, and pain at a 200–250 mg intravenous dose on day 1 every 3 weeks, another clinical trial is continuing with relapsed or refractory acute myeloid leukemia and a small-cell lung cancer [11, 39]. The recent study has suggested that BI 2536 is also an inhibitor of a new family of bromodomains, a novel drug target for cancer [33]. These collective findings suggest that BI 2536 might be a better and more effective therapeutic agent, in addition to its minimum toxicity and promising selectivity throughout clinical development.

### 2.8. BI 6727 (Volasertib)

BI 6727, which was also developed by Boehringer Ingelheim, is another PLK-1-selective ATP-competitive inhibitor in the dihydropteridinone series with improved properties. It exhibits inhibition activity against the targeted catalytic domain of PLK-1/2/3 at different half-inhibitory concentrations of 0.85, 5.0, and 56 nM, respectively. The inhibitory potential of BI 6727 has been screened against a wide range of tumor cell lines* in vitro*. BI 6727-treated cells experienced G2/M phase arrest, along with a polo-like spindle resemblance phenotype and subsequently induced apoptosis [34]. BI 6727 exhibits strong efficacy against not only standard nude mouse xenografts models of human NSCLC and a taxane-resistant CXB1 model of colorectal cancer but also colon, pancreatic, and breast cancer. It showed good oral availability during the study of absorption, distribution, metabolism, excretion (ADME), and toxicity analysis. Furthermore, the toxicity level of BI 6727 was well-tolerated in a phase I study with patients with advanced or metastatic solid tumors using a 12–450 mg dose [35]. However, BI 6727 caused reversible neutropenia and thrombocytopenia during the clinical trial. BI 6727 was also well-tolerated during two dosing schedules of phase I clinical trials with advanced solid tumor malignancies. The MTD carried out on schedule A was 300 mg and 150 mg for schedule B, and the DLT of BI 6727 were reversible neutropenia, febrile neutropenia, and thrombocytopenia [40]. Moreover, BI 6727 is now part of a phase II clinical trial as a monotherapy agent, as well as in combination with pemetrexed compared with pemetrexed alone in advanced NSCLC (https://clinicaltrials.gov/ct2/show/study/NCT00824408). BI 6727 phase II clinical trials are also ongoing with platinum-resistant/refractory ovarian cancer (http://meetinglibrary.asco.org/content/112794-132). In addition, BI 6727 has been tested with Aurora kinase inhibitor BI 811283 against pediatric malignancy cell lines, including Ewing sarcomas, leukemias, medulloblastomas, neuroblastomas, and osteo- and rhabdomyosarcomas* in vitro*. In this study, it was observed that BI 6727 showed a GI_50_ ranging from 2.89 nM/L to 5.05 nM/L and also showed G2/M arrest at 24 hr, leading to apoptotic cell death at 48 hrs in an RMS-1 xenograft model of rhabdo blastoma in pediatrics tumors [41].

### 2.9. Cyclapolin1

Cyclapolin1 is a selective, non-ATP-competitive type inhibitor of PLK-1, developed by Cyclacel Ltd., Cambridge, UK. Cyclapolin is a benzthiazole-N-oxide derivative that is currently undergoing preclinical evaluation to improve its efficacy with minimum toxicity [7]. Cyclapolin1-treated cells showed a spindle collapse phenotype in the human and* Drosophila* cell lines HeLa and S2 cells, respectively. Moreover, Cyclapolin1 reduced the centrosome integrity and microtubule nucleation ability. Several cancer cell lines have been screened against cyclapolin1 to evaluate the potential for an IC_50_ of ~20 nM [42].

### 2.10. DAP-81

DAP-81 is a diamino-pyrimidine derivative designed at Rockefeller University, New York. DAP81 destabilized the kinetochore-microtubule assembly, whereas other spindle tubules are stabilized, leading to the monopolar spindle phenotype [43]. DAP-81 inhibits PLK-1 with an IC_50_ of 0.9 *μ*M and shows the polo-like phenotype resemblance. Moreover, DAP-81 has also been proven to be an inhibitor by reducing the phosphorylation of CDC25C in a dose-dependent manner [43]. DAP-81 is now ongoing in preclinical evaluation to enhance efficacy and reduce toxicity [35].

### 2.11. ZK-Thiazolidinone (TAL)

TAL is an ATP-competitive type inhibitor of PLK-1 designed by Bayer Schering Pharma AG, Berlin, Germany. TAL inhibits the activity of the catalytic domain of PLK-1 with an IC_50_ of 19 ± 12 nM* in vitro* [44]. A number of human and mouse cancer cell lines have been screened to evaluate the potential of TAL and exhibited an inhibitory effect at an IC_50_ of 0.2–1.3 *μ*M. TAL-treated cells exhibited mitotic G2/M arrest and showed monopolar-phenotypic resemblance, along with apoptosis. Moreover, TAL-mediated inhibition also participated in impairment of centrosome maturation and lead to the failure of cytokinesis [44]. Preclinical evaluation of TAL is ongoing to improve its efficacy and reduce the toxicity level. Moreover, TAL also synergizes its inhibitory effect in combination with other antitumor agents.

### 2.12. PHA-680626

PHA-680626 is a pyrrolo-pyrazole derivative, and it inhibits PLK-1. Initially, it was developed as an Aurora-A inhibitor with an IC_50_ of 0.07 *μ*M [15]. Later, it observed that PHA-680626 also inhibited PLK1/2/3 at an IC_50_ of 0.53/0.007/1.67 *μ*M* in vitro*, respectively [15]. PHA-680626 is now currently undergoing preclinical trials.

### 2.13. SBE13

SBE13 is a vanillin derivative, and it inhibits PLK-1 with an IC_50_ of 12–39 *μ*M [45]. SBE13 showed a 1000-fold greater inhibition selectivity among PLK family members, and a wide range of cancer cell lines have been screened to evaluate the anti-proliferative effect of SBE13, and it caused G2/M arrest at higher concentrations (http://www.tandfonline.com/doi/pdf/10.4161/cc.9.3.10721). SBE13 is currently undergoing a preclinical phase of development [45].

### 2.14. Compound 36

Compound 36 belongs to the imidazopyridine class of inhibitors, and is selective for the inhibition of PLK-1. Compound 36 exhibited high selectivity against PLK-1 among a panel of 212 kinases [46]. Compound 36 showed 2-fold and 18-fold greater inhibition selectivity to PLK-1 compared to PLK family members PLK-2 and PLK-3, respectively. Furthermore, Compound 36 inhibits PLK-1 with an IC_50_ of 9.8 nM* in vitro*, whereas it inhibited the other PLK family members, PLK-2 and PLK-3 with an IC_50_ of 21 nM and 178 nM, respectively. Compound 36 is also currently undergoing preclinical development [46].

### 2.15. LFM-A13

LFM-A13 is a leflunomide metabolite analog and a selective inhibitor of PLK-1 [47]. It inhibits the purified recombinant Plx1 (Xenopus homolog of human PLK-1) with an IC_50_ of 32.5 *μ*M, whereas inhibits PLK-3 with an IC_50_ of 61 *μ*M* in vitro*. On the basis of molecular docking studies, it has been predicted that LFM-A13 binds to the catalytic site of the Plx1 kinase domain by using a GST-CDC25 substrate peptide interaction. LFM-A13-treated cells exhibited mitotic arrest and prevented bipolar spindle assembly formation in human cancer cell lines, including breast cancer and glioblastoma. Furthermore, LFM-A13 showed tolerable toxicity in mouse and rat tumor models, without hematological toxicity such as peripheral blood counts and bone marrow suppression [47]. Moreover, another study demonstrated that LFM-A13 also showed antileukemic activity against human leukemic B-cell precursors [48]. In addition, several xenografts in NOD/SCID mouse studies demonstrated that LFM-A13 did not show notable levels of adverse effects in alone or in combination* in-vivo*. Furthermore, the combined study of LFM-A13 with the antileukemic drug vincristine, exhibited inhibitory activity against PLK-1 without any sign of morbidity or mortality. The preclinical studies of LFM-A13 support the hypothesis that chemoresistance of relapsed B-cell precursor acute lymphoblastic leukemia can be cured by using LFM-A13 in combination with vincristine. LFM-A13 continues ongoing preclinical development [48].

### 2.16. RO3280

RO3280 is a derivative of the pyrimidodiazepines series and is recognized as a PLK-1 inhibitor [49]. RO3280 inhibited the enzymatic activity of PLK-1 with an IC_50_ of 3 nM and EC_50_ of 6.0–82 nM against several cancer cell lines* in vitro*. Furthermore, RO3280 has also been screened against a wide panel of 318 kinases (wild type and mutant) to evaluate its selectivity and potency. The binding potential of RO3280 to PLK-1 is 500-fold tighter than any other protein kinase in the panels. Moreover, RO3280 also showed higher efficacy against a series of mouse xenograft models. Additionally, RO3280 exhibited robust antitumor activity at a dose 40 mg/kg/week and reduced 72% of tumor growth, whereas at higher dose of RO3280 reduced tumor growth completely* in vivo *[49]. RO3280-treated leukemic cells exhibited apoptosis induction and cell cycle disorder caused by the inhibition of Bcl-2, BTK, and CASP1 [50].

### 2.17. Compound 15

This compound is a derivative of a novel 2-amino-isoxazolopyridine and inhibits PLK-1 activity with an IC_50_ of 0.051 *μ*M [51]. Compound 15 is a selective ATP-competitive type inhibitor of PLK-1* in vitro*. However, compound 15 also inhibited other members of the PLK family, namely, PLK-2/3, with an IC_50_ of 0.172 *μ*M and 1.382 *μ*M, respectively. Compound 15-treated HCT116 colorectal carcinoma cell lines displayed monopolar spindles phenotype resemblance and mitotic arrest, which induced apoptosis [51].

### 2.18. Compound 38

This compound is a derivative of 2-amino-pyrazolopyridine and is identified as a selective ATP-competitive type inhibitor of PLK-1 [52]. Compound 38 inhibited PLK-1with an IC_50_ of 0.042 *μ*M and an EC_50_ of 3.64 *μ*M* in vitro*. Compound 38-treated HCT116 colorectal cancer cell line exhibited G2/M cell cycle arrest and lead to apoptosis. In addition, Compound 38 showed 50-fold more inhibition selectivity to PLK-1 over PLK-2 and PLK-3 [52].

### 2.19. TAK-960 (4-((9-Cyclopentyl-7,7-difluoro-5-methyl-6-oxo-6,7,8,9-tetrahydro-5H-pyrimido(4,5-b)(1,4)diazepin-2-yl)amino)-2-fluoro-5-methoxy-N-(1-methylpiperidin-4-yl)benzamide)

TAK-960 is a novel, potent, orally bioavailable and selective ATP-competitive inhibitor of PLK-1 [53]. TAK-960 inhibits PLK-1 with an IC_50_ of 0.8 nM and the other PLK family members, namely, PLK-2 and PLK-3, with an IC_50_ of 16.9 nM and 50.2 nM* in vitro, *respectively [53]. Moreover, TAK-960 has been screened against a wide panel of 282 human kinases without showing notable activity against any kinase of the panel* in vitro*. Furthermore, TAK-960 was also evaluated against a wide range of cancer cell lines with a status of mutation in* TP53* and* KRAS*, including multidrug-resistant cell lines (MDR1), and exhibited an EC_50_ in the range of 8.4 to 46.9 nM. TAK-960-treated HT-29 colorectal cancer cells displayed a monopolar spindle phenotype, and the cells were arrested in the G2/M phase, subsequently lead to apoptosis. Furthermore, TAK-960 inhibited a wide range of tumor xenograft models by using a dose of 10 mg/kg once daily for 2 weeks, which was selected on the basis of experiments with HT-29 colorectal cancer xenografts. In addition, TAK is well-tolerated an oral dose of 7.5 mg/kg once daily for 9 days and showed significant growth inhibition for an MV4-11 human leukemia model* in-vivo *[53]. TAK-960 is no longer in clinical trials due to a lack of the efficacy during development (https://clinicaltrials.gov/ct2/show/results/NCT01179399).

### 2.20. TKM-080301

TKM-080301, which was developed by Tekmira Pharmaceuticals Corp., is a lipid nanoparticle- (LNP-) based formulation of a (si)RNA that targeting human PLK-1 mRNA and has shown not only potent antiproliferative activity and gene-specific silencing against many human cancer cell lines but also potent antitumor activity against several xenograft models of human cancers. TKM-080301 is a highly selective inhibitor for PLK-1 without affecting the remaining members of the PLK-1 family. Moreover, it also demonstrated potential antitumor activity in implanted xenografts intrahepatically and subcutaneously. TKM-080301-mediated silencing of PLK-1 mRNA also persisted for up to 7–10 days/single administration without any notable stimulation of the innate immune response. TKM-080301 is highly restricted to the liver and spleen because the toxicity profile of TKM-080301 was governed by the LNP distribution. In a phase I study, the estimated MTD of TKM-080301 is 0.75 mg/Kg against various types of human cancers with hepatic metastases and showed tolerated side effects, including fever, rigors pyrexia, chills, nausea, vomiting, and fatigue. Moreover, TKM-080301 was well-tolerated at the MTD in a phase I study, and phase II study is currently underway. TKM-080301 has been administered intravenously against specific neuroendocrine tumors (NET) and adrenocortical carcinoma (ACC) (Mark Kowalsk et al. https://clinicaltrials.gov/ct2/show/NCT01262235, February 20, 2015).

### 2.21. Targeting against the PLK-1 Polo Box Domain (PBD)

The major disadvantages to blocking the kinase activity of PLK-1 are that ATP-competitive inhibitors commonly inhibit all PLK paralogs, including PLK-3 (tumor suppressor), and a single point mutation in the catalytic site of PLK-1 (S67V) results in dramatic resistance to the structurally diverse ATP-competitive inhibitors [54]. The PBD of each PLK-1 helps to determine the substrate recognition, mitotic PLK-1 activation, and subcellular localization. Moreover, PBD-substrate interaction facilitates the activation of PLK-1 in mitotic phase, which phosphorylates the bound substrate and targets the mitotic structure for cell cycle progression. Temporal-spatial subcellular localization, mitotic activation, and progression-related studies have proven that the PBD is an essential component of PLK-1, and the inhibition of the PBD induced a monopolar spindle appearance similar to catalytic inhibition of PLK-1 [11, 55]. During the last 5 years, several drug discovery studies observed that the PBD is an attractive, alternative drug target for the development of PLK-1 inhibitors [56–59]. Furthermore, an examination of the crystal structure of the PBD-phosphopeptide interaction illustrated that Trp414, His538, and Lys540 residues of PBD play an important role in the inhibition of PBD-dependent protein-protein interactions without affecting ATP binding. Moreover, small molecules and phosphopeptides have been identified to mimetic the PBD and inhibit subcellular localization of PLK-1 [60, 61]. In addition, study of the PBD-phosphopeptide complex also facilitates the structure-based optimization of lead compounds to identify a PBD-dependent inhibitor of PLK-1 [62]. Moreover, phosphopeptide Pro-Leu-His-Ser-p-Thr- (PLHSpT-) treated cells have induced G2/M arrest and apoptosis-mediated cell death in cancer by inhibiting the PBD of PLK-1* in vitro* and* in vivo* as shown in Table 2 [63].

### 2.22. Poloxipan

Poloxipan is a pan-specific inhibitor of the PBD that was isolated through the screening of chemical libraries. Poloxipan inhibits the PBD of PLK-1/2/3 with an IC_50_ of 3.2 ± 0.3 *μ*M, 1.7 ± 0.2 *μ*M and 3.0 ± 0.1 *μ*M, respectively, along with the inhibition of other phosphoserine/phosphothreonine-binding domains, such as the forkhead-associated (FHA) domain of CHK-2, WW domain of peptidyl-prolyl cis/trans isomerase (PIN1) and phosphotyrosine-binding domains (SH2 domains of STAT1/3/5 and lymphocyte-specific protein tyrosine kinase) [72].

### 2.23. Poloxin

Poloxin is a synthetic derivative of a well-known PBD antineoplastic inhibitor named thymoquinone, which was identified using a fluorescence polarization-based high-throughput screening assay against a wide library of 20,000 small molecules. Poloxin showed a better specificity profile in comparison to its parent molecule. Poloxin inhibited the PBD with an IC_50_ of 4.8 ± 1.3 *μ*M* in vitro* and* in vivo *[57]. Furthermore, poloxin also inhibited other members of the PLK family, including PLK-2/3 with an IC_50_ of 18.7 ± 1.8 *μ*M and 53.9 ± 0.8 *μ*M, respectively. Moreover, poloxin-treated cells showed similar phenotypic appearances, such as catalytic inhibition of PLK-1 [57]. Furthermore, another study showed that poloxin may interact covalently with a nucleophilic Cys residue present in the nearby SpT pocket of PBD [79]. The recent study highlighted that poloxin and its parental molecule thymoquinone interact with the pSer/pThr binding pocket of PBD, and permitting the small molecule intervention of the phosphorecognition of PLK-1 by a phosphate mimic [70].

### 2.24. Purpurogallin (PPG)

PPG is a natural benzo tropolone compound derived from nutgall and oak bark. PPG was identified after high-throughput screening of 2500 natural compound repositories at RIKEN Saitama, Japan. PPG inhibits the PBD with an IC_50_ of ~0.3 *μ*M in a GST-pull down assay using the PBD as the target for WEE1* in vivo *and* in vitro*. Moreover, PPG also inhibits PLK-2 but not PLK-3 [71]. Furthermore, PPG also inhibits the tyrosine-specific protein kinases human immunodeficiency virus 1 integrase and prolyl endopeptidase, as determined by a screen of a wide panel of kinases. The inhibitory concentration of PPG is higher for Bcl_-XL_-BH3 and BAD inhibition in comparison to PLK-1. Moreover, PPG-treated cells exhibited prolonged progression of mitosis and delay its onset. PPG-treated cells did not show the monopolar spindle like appearance, but exhibited improper chromosome alignment [71]. In addition, a structure-activity relationship (SAR) analysis showed that the 4-hydroxyl group of PPG is an essential entity for the inhibition of PBD [71].

### 2.25. Aristolactam AIIIa

This compound is a derivative of the natural compound Aristolactam, which was isolated from a natural product library by random screening against the PBD of PLK-1 [73]. Aristolactam AIIIa-treated cells exhibited antiproliferative activity and induced mitotic arrest with spindle abnormalities. Furthermore, Aristolactam AIIIa also inhibited the drug resistance cell line HCT-8/V. Surprisingly, Aristolactam AIIIa not only targets the PBD but also shows robust catalytic inhibition of PLK-1 compared to previously published inhibitors of PBD, with an IC_50_ of 47.5 *μ*M* in vitro. *Moreover, Aristolactam AIIIa also inhibited PBD-dependent binding with an IC_50_ of 10 *μ*M in an SBR assay [73].

### 2.26. Phosphopeptides as a PBD Inhibitor

PLK-1 was identified as a cell cycle-regulating mitotic kinase because the inhibition of PLK-1 caused the polo-like structure of the spindle fiber in the nucleus. The PBD domain is identified as a chemotherapeutic target for cancer based on its implication in PBD-dependent substrate targeting and subcellular localization of PLK-1. PBD has an evolutionarily conserved phosphoserine/phosphothreonine motif in humans,* Xenopus,* and yeast. The crystal structure of the PBD-phosphopeptide complex shows that the phosphopeptide binds within a positively charged cleft located at the edge of PBD interface. This cleft is essential for PLK-1 to recognize the substrate and to regulate PLK-1 [60]. A loss of function study observed that site-directed mutation in the positively charged cleft leads to disruption of phosphodependent interaction and subcellular localization of PLK-1 both* in vitro *and* in vivo*. Therefore, these studies collectively provide robust evidence that PBD-phosphopeptide binding is essential for PLK-1 targeting to recognize the substrate and also regulate the PLK-1. The inhibition of PBD leads to cell cycle arrest, inducing the apoptosis [60].

### 2.27. MQSpTPL and PMQSpTPL

This inhibitory peptide was designed after a study of the phosphopeptide-PBD complex. MAGPMQSpTPLNGAKK (Poloboxtide) was the first designed phosphopeptide, and it was successfully used in various biochemical assays. Poloboxtide inhibited the enzymatic activity of PLK-1 with an IC_50_ of 5 *μ*M in a dose-dependent manner* in vitro*. Furthermore, MQSpTPL phosphopeptide is also recognized by the pincer grip-like pockets of PB1 and PB2, which are formed by Lys540 and His538 residues. These residues directly interact with the phosphate group of the phosphopeptide [61, 74]. Moreover, a side-chain of the Ser-containing phosphopeptide forms a hydrogen bond with a highly conserved Trp414 residue in all PBDs. In addition, a loss-of-function study observed that a mutation in PBD Trp414-Phe disrupts the subcellular localization of PLK-1 to the spindle poles and abolishes the function of PLK-1 [55].

### 2.28. LLCSpTPNG

This phosphopeptide was designed on the basis of the crystal structure of PBD-phosphatase CDC25C complex. LLCSpTPNG is recognized by the Trp414 residue of PB1. Moreover, loss-of-function studies revealed that the mutation of Trp414-Phe abolished the molecular recognition and subcellular localization of PLK-1to the centrosome [61, 75].

### 2.29. PLHSpT

This optimal phosphopeptide was designed on the basis of the molecular interaction of PBD and PBD-binding protein-1(PBIP1) with a high level of binding affinity and specificity via the PBIP1-p-T78 motif [80]. Furthermore, PBD-phosphopeptide complex analysis revealed that an N-terminal Pro-residue plays a critical role in ensuring specificity for Trp414, Phe535, and Arg516 residues of PBD [80]. PLHSpT inhibited the PBD in a dose-dependent manner with a K_D_ of 0.445 mM. Moreover, the phosphatase-resistant pThr mimetic (2S, 3R)-2-amino-3-methyl-4-phosphonobutyric acid- (Pmab-) containing peptide PLHS-Pmab bound to the PBD without reducing the affinity and specificity of the original peptide [76]. As a consequence, HeLa cells transfected with PLHSpT-Pmab exhibited mitotic arrest. These collective findings have documented that small phosphopeptide-based specific inhibition of PLK-1 PBD can be feasible [76].

## 3. Conclusion

The essentiality of PLK-1 for normal cell cycle progression and mitosis has raised lingering questions regarding the targeting value of PLK-1 in cancer chemotherapy [11, 81, 82]. Many anticancer targets that are strongly implicated in cancer progression and tumor growth have been identified. Small molecule inhibitors for PLK-1 showed substantial inhibitory potential against tumor and induced the apoptosis. However, these inhibitors also show a high load of adverse effects, including bone marrow suppression, neutropenia, and heart disease. Many of the cancer targets are proto-oncogenes and are implicated in the normal cell cycle progression. Therefore, the main point of focus is that the small molecule inhibitor should display maximum selectivity and molecular specificity against targets in addition to validation of the essentiality of the target for normal cells in order to minimize side effects. Although, these inhibitors show good inhibitory potential to reduce tumor growth* in vivo* and* in vitro*, they also inhibit other vital protein kinases due to their similarity in the highly conserved catalytic domain. These inhibitors, therefore, inhibit the catalytic activity of targeted protein along with nontargeted proteins. Thus, these inhibitors should be validated against the 518 protein kinases of the human kinome to minimize adverse effects and maximize the selectivity during treatment or clinical trials.

PLK-1 is the most promising mitotic kinase validated as a chemotherapeutic cancer target because PLK-1 is critically essential for cancer cell survival and not for normal cells. The overexpression of PLK-1 is actively implicated in the failure of mitotic checkpoint arrest and tumor progression. Several studies collectively have proven that the (si)RNA-mediated severe inhibition of PLK-1 reduced tumor size without affecting the viability of normal cells [83]. Therefore, this work provides clear-evidence that PLK-1 is exclusively considered as the most promising cancer drug target with minimal adverse complications. Numerous studies have been published examining the potential of PLK-1 as an antitumor drug target, including work with antisense oligonucleotides, small interfering (si)RNA, and small molecules with reversible and tolerable cytotoxicity, and it has also been proven that severe depletion of PLK-1 does not affect the normal cell growth [31, 84, 85]. Many PLK-1 inhibitors, such as BI 2536, are currently under study in different phases of clinical trials with well-tolerated toxic effects and show a good efficacy profile with the more than a 1-fold selectivity index against the other PLK family members. Therefore, these collective findings provide documentation that the significant reduction in overexpression of PLK-1 by small molecules, (si)RNA, antisense oligonucleotides, and phosphopeptides diminish the viability of cancer cells without affecting the viability of normal cells [83, 86]. Hence, PLK-1 is the most efficient, validated drug target to inhibit cancer growth with the maximum selectivity index.

## Figures and Tables

**Figure 3:**
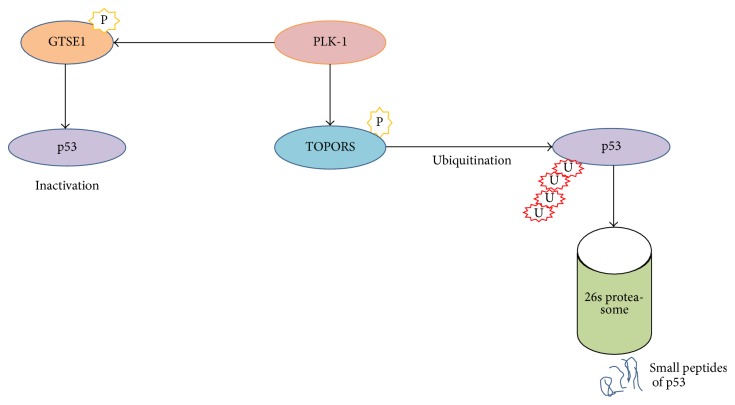
Model of regulation the Topo-1 binding protein- (TOPORS-) mediated degradation of p53 by PLK-1 in the response of long time cell cycle arrest. PLK-1-mediated phosphorylation of TOPORS at Ser-718, leads the ubiquitination of p53 to proteasomal degradation. PLK-1 mediated phosphorylation of GTSE1 inactivates the tumor suppressor gene p53. Consequently, PLK-1-mediated inactivation and/or degradation of p53 causes the tumorigenesis.

**Table 1:** PLK-1 kinase domain-targeted inhibitors.

Compounds	Chemical class	Synonyms	IC_50_ values for PLK-1^a^	Mechanism of action	Selectivity	Selectivity index *X* = b/a, *Y* = c/a, *Z* = d/a	Interacting residues	References
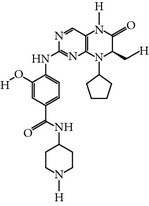 BI 2536	A dihydropteridinone developed by Boehringer Ingelheim	UNII-4LJG22T9C6, BI-2536	0.83 nM	ATP-competitive inhibitor	(i) Exhibited 1,000-fold selectivity against a wide panel of tyrosine and serine/threonine kinases (ii) PLK2^b^ IC_50_ = 3.5 nM (iii) PLK3^c^ IC_50 _= 9.0 nM (iv) EC_50_ = 2–25 nM	*X* = 4.21-fold *Y* = 10.84-fold *Z* = ND	Cys133, Leu132, Leu59, Arg136, Arg57, Glu140, Cys67, Lys82, Ala80, Leu130, Gly60, Phe183, Asp194, Val114 PDB ID: 2rku (hPlk1 KD 13–345, T210V)	[11, 16, 31, 64]

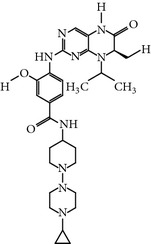 BI 6727	A dihydropteridinone developed by Boehringer Ingelheim	Volasertib (USAN), UNII-6EM57086EA	0.87 nM	ATP-competitive inhibitor	(i) No inhibitory activity against a wide panel of more than 50 protein kinases (ii) PLK2 IC_50_ = 5 nM (iii) PLK3 IC_50_ = 56 nM (iv) EC_50_ = 11–37 nM	*X* = 5.7-fold *Y* = 64.36-fold *Z*-ND	Cys133 (hPlk1 Kinase domain 13–345, T210V) PDB ID: 3fc2	[34, 65]

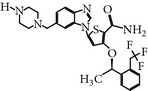 GSK 461364A	Thiophene benzimidazole developed by Glaxo SmithKline	UNII-8QO27TK6Q4, GSK-461364, 2-Thiophenecarboxamide, 5-(6-((4-methyl-1-piperazinyl)methyl)-1H-benzimidazol-1-yl)-3-((1R)-1-(2-(trifluoromethyl)phenyl)ethoxy)-	2 nM	ATP-competitive Inhibitor,	Has 400-fold greater potency for PLK1 than for PLK2 and PLK3, EC_50_ < 50 nM in > 83% of the 120 cancer cell lines tested	*X* and *Y* = 400-fold *X* = ND	Glu140 (Homology Model)	[24–27, 66]

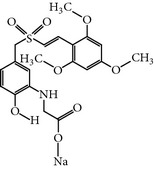 ON01910.Na	Undefined by Onconova Therapeutics	Rigosertib sodium'Novanex, UNII-406FL5G00V, Estybon	9-10 nM,	A non-ATP-competitive Plk1inhibitor; Affects microtubule dynamics	Also inhibits PDGFR, ABL, FLT1, CDK-2, PLK-2, Src, and Fyn. Efficacious both as a single agent and in combination with cytotoxic drugs in xenograft models	ND	ND	[11, 14, 15, 67]

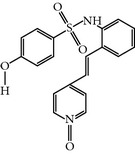 HMN-176	Stilbazole compound by Nippon Shinyaku Co. Ltd.	(E)-4-((2-N-(4-Methoxybenzenesulfonyl)amino) stilbazole)1-oxide	118 nM	ATP-competitive inhibitor	Shows potent antitumor activity in gastric, breast, and lung human tumor xenografts and so forth. Better activity compared to known drugs such as cisplatin, doxorubicin, vincristine, and tegafur-uracil Inhibits the expression of NF-Y and induces the cell cycle arrest	ND	ND	[19, 21, 22]

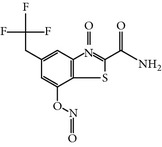 Cyclapolin 1	Benzthiazole-3-oxide derivative developed by Cyclacel Ltd., Cambridge, UK	Calthor, Citosarin, Cyclapen, Noblicil, Orfilina, Ultracillin, Cyclapen-W, Vastcillin,Vipicil,Wypicil Ciclacillinum, Cyc-800	20 nM	Noncompetitive with respect to ATP	Inhibits PLK1; other family members were not determined Inhibits C-terminal Src kinase; IC_50_~100 *µ*M Cell cycle may also be affected in G1/S	ND	ND	[42]

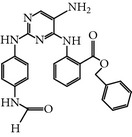 DAP-81	Diaminopyrimidine Derivative by R0ckefeller University, New York	Dialkylphthalate-810, DAP-810	0.9 nM	Predicted to target the nucleotide pocket	Destabilized kinetochore microtubules. Dose-dependent reduction of CDC25C phosphorylation in cells and recapitulation of key aspects of the loss-of-function phenotype for PLK1	ND	ND	[43]

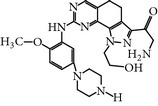 NMS-P937	Pyrazolequinazoline by Nerviano Medical Science	UNII-67RM91WDHQ NMS-1286937	20 nM	ATP-competitive inhibitor	More than 100 cell lines and 200 protein kinases have been tested Shows prolonged M phase and induce apoptosis Active in Xenograft tumor model IC_50_ < 100 nm on solid tumor	ND	Giu131, Cyc133, Lys82, Asp194, Cys67, Phe183, Arg57, Leu132-Cys133-Arg134	[20, 23, 29]

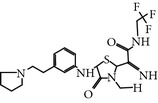 ZK-Thiazolidinone	Developed by Bayer Schering Pharma AG, Berlin, Germany	TAL	19 ± 12 nM	ATP-competitive Inhibitor	Induced arrest in prometaphase-like arrest and finally cytokinesis failure and multinucleation IC_50_ = 0.2–1.3 *μ*M on human and mouse tumor cell lines	ND	ND	[44]

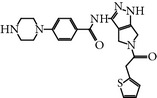 PHA-680626	Pyrrolo-pyrazole derivative	NA	0.53 nM	ND	PLK-2 (IC_50_ = 0.07 *µ*M) PLK-3 (IC_50_ = 1.61 *µ*M) Weaker inhibition was detected on few kinases	*X* = 132-fold *Y* = more than 3000-fold	Glu131, Cys133, Lys82, His105 PDB ID: 2owb (hPlk1 KD, T210V)	[15]

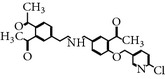 SBE13	Vanillin derivative	NA	EC_50_ = 12–39 *µ*M	ND	Shows 1000-fold selectivity within the PLK family	*X* or *Y* or *Z* = 1000-fold	Arg93, Asp194, Cys133, Phe195, Phe183 (By homology model)	[45]

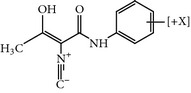 LFM-A13	*α*-cyano-*β*-hydroxy-*β*-methyl- *N*-(2,5-dibromophenyl)-propenamide	NA	Plx1 32.5 *µ*M using GST-CDC25 as a substrate		PLK-3 IC_50 _= 61 *µ*M Also inhibits human BTK with an IC_50_ of 17.2 ± 0.81 *µ*M The activity is 3–15 fold greater against a panel of protein kinases	ND	ND	[47, 48, 68]

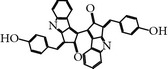 Scytonemin	Subunit derived from tryptophan and Phenylpropanoid isolated from many strains of cyanobacteria.	(3,3′-Bis((4-hydroxyphenyl)methylene)-(1,1′-bicyclopent(b)indole)-2,2′(3H,3′H)-dione)	2.0 ± 1 *µ*M	ATP-competitive Inhibitor	Also inhibits the transcriptional factor MYT1 CDK-1, Chk-1, and PKC Does not directly inhibit PLK-1 up to 3-4 *µ*M	ND	ND	[7, 12]

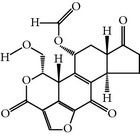 Wortmannin	Steroidal furanoids, originally isolated from Penicillium wortmannii	BRN 0067676, NSC 627609, SL-2052, UNII-XVA4O219QW, Wartmannin	24 nM	ATP-competitive Inhibitor	Also inhibits the other member of PLK family and interacts with similar binding affinity Inhibits the PI3K	ND	Lys68 Cys119 PDB ID: 3d5x (zebrafish Plk1 kinase domain, 1–312 wild type and 13–312 T196D)	[13, 17, 18]

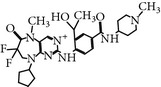 RO3280	Pyrimidodiazepines derivatives	NA	0.09 nM	ATP-competitive Inhibitor	318 wild type and mutants protein kinases tested More than 85% protein kinases inhibits at 1 mM	500- greater binding affinity with PLK-1 compared to tested penal of protein kinases	ND	[49]

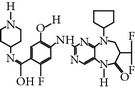 TAK-960	[4-[(9-cyclopentyl-7,7-difluoro-5-methyl-6-oxo-6,7,8,9 tetrahydro-5H pyrimido[4,5-b][1,4]diazepin-2-yl)amino]-2-fluoro-5-methoxy-N-(1-methylpiperidin-4-yl) benzamide]	TAK 960,	0.8 nM	ATP-competitive Inhibitor	No inhibitory activity against 282 protein kinases Anti-tumor activity against *TP53, KRAS, MDR *mutated cell lines Monopolar spindle and G2/M phase arrest	*X* = 21-fold *Y* = 62-fold *Z* = ND	ND	[53]

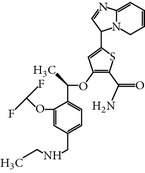 Compound 36	Imidazopyridine derivative	NA	9.8 nM	ATP-competitive Inhibitor	No inhibitory activity against 212 protein kinases at 1 *µ*M. Tolerated toxicity observed against WBC	*X* = 2-fold *Y* = 18-fold *Z* = ND	Cyc133, Lys82, Asp194	[27, 46]

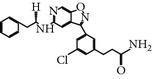 Compound 15	2-Amino-isoxazopyridine	NA	0.051 *µ*M	ATP-competitive Inhibitor	Treated cells showed monopolar phenotype and mitotic arrest in colorectal carcinoma cell lines	*X* = 3.37-fold *Y* = 27-fold *Z* = ND	ND	[51]

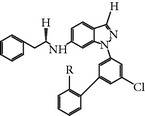 Compound 38	Derivative of 2-amino-pyrazolopyridines	NA	0.042 *µ*M	ATP-competitive Inhibitor	HCT116 colorectal cancer cell lines showed G2/M arrest and induced apoptosis	*X* and *Y* = 50-fold *Z* = ND	Phe169, Cys53, Cys119, Lys68 (hPlk1Lys82), Asp180 (hPlk1Asp 194), PDB ID: 3dbc (zPlk1KD, T196D)	[52]

TKM-080301 RNAi	Lipid nanoparticle based formulation developed by Tekmira Phaarmaceuticals Corp.	NA	ND	Silencing PLK-1 mRNA	Showed antiproliferative and gene silencing activity against human cancer cell lines Antitumor activity against human cancer xenografts	ND	PLK-1 mRNA silencing	[69]

a = PLK-1, b = PLK-2, c = PLK-3, d = PLK-4, ND = not determined, PLK = polo-like kinase, IC_50_ = half-maximal inhibitory concentration, EC_50_ = effector concentration for half-maximum response, BTK = Bruton's tyrosine kinase, PBD = Polo box domain, NIMA-interacting 1, Plx1 = *Xenopus *homologue of PLK-1, MYT1 = myelin transcription factor 1, PDGFR = platelets derived growth factor receptor, ABL = Abelson murine leukemia viral oncogene homolog 1, FLT1 = vascular endothelial growth factor receptor 1, CDK-1/2 = cyclin-dependent kinase-1/2, PKC = protein kinase C, PI3K = phosphoinositide-3-kinase, KRAS = Kirsten rat sarcoma viral oncogene homolog, TP53 = tumor suppressor p53, NF-Y = nuclear transcription factor Y subunit alpha, CDC25C = M-phase inducer phosphatase 3, Chk-1 = checkpoint kinase-1, and NA = Not available.

**Table 2:** PLK-1 PBD domain targeted inhibitors.

Compounds	Chemical class	Synonyms	IC_50_ value for PLK-1-PBD^a^	Mechanism of action	Selectivity	Selectivity index *X* = b/a, *Y* = c/a, *Z* = d/a	Interacting residues	References
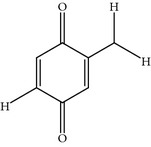 Thymoquinone	Isolated from *Nigella sativa *	EINECS 207-721-1, NSC 2228, p-Cymene-2,5-dione, Thymoquinon, Thymoquinone	1.14 ± 0.4 *µ*M	Interrupt the PLK-1-PBD interaction *in vitro *and *in vivo *	Interacting with polo box domain and interrupt subcellular localization of PLK-1 Also inhibits phosphoser/phosphothr Chk2 FHA domain, Pin1 WW domain, phosphotyr binding src homology 2 (SH2) domain of STAT3	ND	Cys544, Arg500, Pro545, Leu546 (Homology Model)	[27, 57, 70]

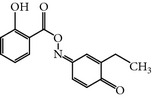 Poloxin	Synthetic derivative of well-known PBD- inhibitor thymoquinone	Poloxime	PLK-1 PBD: 4.8 ± 1.3 *µ*M	Interferes with PLK-1-PBD functions *in vitro *and *in vivo *	Poloxin inhibits other subtypes of the phosphothr/phosphoser binding domains (FHA domain of Chk2, WW domain of PIN1) and the phosphotyr-binding domains (SH2 domains of STAT1, STAT3, STAT5 and LCK), similar phenotype like PLK-1 ATP competitive inhibitor	*X* = 3.8-fold *Y* = 11.22 fold *Z* = ND	Cys544, Arg500, Pro545, Leu546, Asn527, Arg507 (Homology Model)	[27, 57]

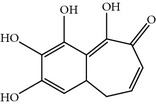 Purpurogallin (PPG)	Benzotropolone natural product derived from nutgall by RIKEN, Japan	CCRIS 8139, EINECS 209-324-9, NSC 35676, Purpurogalli, Purpurogalline, UNII-L3Z7U4N28P	0.3 *µ*M in GST-pulldown assays using PLK1 PBD as bait for WEE1A	Inhibits PBD-dependent binding *in vitro *and *in vivo *by 2-hydroxyl group	Also inhibits HIV-1 integrase, tyrosine protein kinases, Bcl-XL, BH3 peptides, prolyl endopeptidases and DNA synthesis of tumor cells Delayed the onset of mitosis Kinetochore localization of CENP-E inhibited, destabilized microtubules interaction	ND	His538, Lys540, Trp 414, Leu491 (Homology Model)	[27, 71]

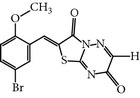 Poloxipan	Panspecific inhibitor	NA	PLK-1-PBD: 3.2 ± 0.3 *µ*M	Inhibits PLK-1-PBD binding manner	Poloxin inhibits other subtypes of the phosphothr/phosphoser binding domains (FHA domain of Chk2, WW domain of PIN1) and the phosphotyr-binding domains (SH2 domains of STAT1, STAT3, STAT5, and LCK)	*X* = 0.53-fold *Y* = 0.93 fold	ND	[72]

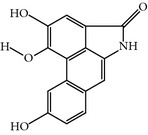 Aristolactam AIIIa	Derivative of natural product Aristolactam AIIIa	CCRIS 2996, Aristolactam-aii, Dibenz(cd,f)indol-4(5H)-one, 2-hydroxy-1-methoxy	PLK-1 = 47.5 *µ*M PBD = 10 *µ*M	Inhibits kinase and PBD domain with different inhibitory concentration	Antiproliferative activity and induced mitotic arrest Inhibits cancer cell lines as well as clinically drug resistance cell line HCT-8/V	ND	ND	[73]

MAGPMQSpTPLGAKK	Optimal phosphopeptide sequence	PoloBoxtide	IC_50_ = 5 *µ*M Kd = 280 nM	PoloBoxtide is recognized by pincer grips like pocket PB1 and PB2	Mutation in PBD trp414-phe disrupts the PLK-1 subcellular localization to spindle poles and abolish the function	ND	Lys540, His538, Trp 414 PDB ID: 1 umw	[55, 60, 61, 74]

LLCSpTPNG and LLCSTPNG Cdc25C-P & Cdc25C	Optimal phosphopeptide derived from Cdc25C protein	NA	Kd = 1.8 *µ*M	LLCSpTPNG is recognized by the trp414 residue of PB1	The loss of function study showed that trp414-phe diminished the molecular recognition and subcellular localization of PLK-1 to the centrosome	ND	Trp414 PDB ID: 2ojs, 2ogq	[75]

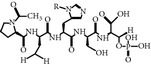 PLHSpT	Minimal phosphopeptide derived from PBIP1 protein for p-T78 motif	NA	Kd = 0.445 *µ*M	The side-chain of N-terminal Pro docked into a surrounding core of hydrophobic amino acid Trp414, Phe535, Arg516 residue	PLHs-Pmab leads the mitotic arrest I HeLa by the inhibition of PLK-1-PBD	ND	His538, Lys540, Trp414, Phe535, Arg 516 PDB ID: 3hik	[41, 76]

a = PLK-1, b = PLK-2, c = PLK-3, d = PLK-4, ND = not determined, PLK = polo-like kinase, IC_50_ = half-maximal inhibitory concentration, EC_50_ = effector concentration for half-maximum response, BTK = Bruton's tyrosine kinase, FHA = forkhead-associated domain, GST = glutathione-*S*-transferase; LCK = lymphocyte-specific protein tyrosine kinase, PBD = Polo box domain, PIN1 = peptidylprolyl *cis*/*trans *isomerase, Plx1 = *Xenopus *homologue of PLK-1, SH2 = Src homology 2, STAT = signal transducer and activator of transcription, CDC25C = M-phase inducer phosphatase 3, Chk-2 = checkpoint kinase-2, NA = not available.

**Table:** (a)

Number	Inhibitors	Cell lines	Xenografts	Animal models	Clinical phase tested cancers	References
1	Scytonemin	Myeloma cells, Jurkat T cells	NA	NA	T-cell leukemia, non-Hodgkin lymphoma	[8, 12]

2	ON01910.Na	HCT-116 colorectal cancer cells, pancreatic cancer cell lines, myelodysplastic syndrome, HL-60, MDS-L, Jurkat, and Ramos, cervical carcinoma	Head and neck squamous cell carcinoma	NA	B-cell chronic lymphocytic leukemia (CLL), brain tumor	[36, 67, 77, 78]

3	Wortmannin	HL-60 cells, MDA-MB-435, MDAMB-231, MCF-7, T-47D, and NCI/ADR	NA	NA	Breast cancer	[17]

4	HMN-176	MDR1 resistant cell lines of ovarian cancer	Gastric, breast, lung human tumor xenograft	Mouse	NA	[21, 22]

5	NMS-P937	Hematological and solid cancer cell lines and <120 cancer cell lines	HCT-116 xenograft tumor model	Rodent and nonrodent	AML, advance and metastatic solid tumor	[20, 29]

6	GSK461364A	120 cancer cell lines, colo205, HT29, A549, MX-1, SKOV-3, HN5, MCF7, N87, PC-3, RKO and so forth	U2OS tumor	Nude mice	Colon, lung, breast, ovarian, colorectal, gastric, prostate, Had and neck squamous cell carcinoma, brain metastasis of brain cancer, non-Hodgkin	[24–26]

7	BI 2536	HeLa	NA	HCT-116 tumor-bearing nude mice	NSCLC, pancreatic, hormone-refractory prostate cancer, relapsed or refractory acute myeloid leukemia and small lung cancer, cervical cancer	[11, 16, 31, 64]

8	BI 6727	Ewing sarcomas, leukemia, medulloblastomas, neuroblasblastoma, osteo- and rhabdomyosarcomas	NSCLC tumor model, Taxane-resistant CXB1 xenograft model of colorectal, RMS-1 xenograft model of rhabdoblastoma in pediatrics tumor	NA	Colon pancreatic and bread cancer, advance or metastatic solid tumor, platinum-resistant/refractory ovarian cancer, pediatric cancer	[34, 65]

9	Cyclapolin 1	Hela	NA	NA	Cervical cancer	[42]

10	DAP-81	NA	NA	NA	NA	[43]

11	ZK-thiazolidinone	Caco2, HeLa, MCF-7	NA	NA	Human and mouse colon, breast, cervical cancer	[44]

12	PHA-680626	NA	NA	NA	NA	[15]

13	SBE13	HeLa	NA	NA	Cervical cancer	[45]

14	Compound 36	NA	NA	HeLa xenograft bearing rats	NA	[27, 46]

15	LFM-A13	Breast and glioblastoma	NA	Mouse and rat tumor models	Human leukemic B-cell precursors	[47, 48, 68]

16	RO3280	H82, H69, colo205, HT-29, MDA-MB-468, PC3	NA	Mouse xenograft model	Leukemic, lung, colon, breast, prostate	[49, 50]

17	Compound 15	HCT-116 Colorectal cancer cell lines	NA	NA	NA	[51]

18	Compound 38	HCT-116 Colorectal cancer cell lines	NA	NA	NA	[52]

19	TAK-960	TP53, KRAS mutated and MDR1 resistant cancer cell lines	NA	HT-29 colorectal cancer xenograft model, MV4-11 human leukemic model	NA	[53]

20	TKM-080301	NA	NA	Implanted xenograft intrahepatically and subcutaneously	Hepatic metastases, specific neuroendocrine tumors and adrenocortical carcinoma	[69]

**Table:** (b)

Number	PBD inhibitors	Cell lines	Mode of action	References
21	Poloxipan	NA	Inhibits PLK-1-PBD binding manner	[72]

22	Poloxin	NA	Interferes with PLK-1-PBD functions *in vitro* and *in vivo*	[27, 57]

23	PPG	NA	Inhibits PBD-dependent binding *in vitro* and *in vivo* by 2-hydroxyl group	[27, 71]

24	Aristolactam AIIIa	HCT-8/V colon resistant cell lines	Inhibit PBD domain ad as well as kinase domain	[73]

25	MQSpTPL	NA	PoloBoxtide is recognized by pincer grip like pocket PB1 and PB2	[55, 60, 61, 74]

26	LLCSpTPNG	NA	LLCSpTPNG is recognized by the trp414 residue of PB1	[75]

27	PLHSpT	NA	The side-chain of N-terminal Pro docked into a surrounding core of hydrophobic amino acid Trp414, Phe535, and Arg516 residue	[41, 76]

NA = not available.
